# Childhood Emotional Neglect and Adolescent Depression: Assessing the Protective Role of Peer Social Support in a Longitudinal Birth Cohort

**DOI:** 10.3389/fpsyt.2021.681176

**Published:** 2021-08-09

**Authors:** Emma A. Glickman, Karmel W. Choi, Alexandre A. Lussier, Brooke J. Smith, Erin C. Dunn

**Affiliations:** ^1^Psychiatric and Neurodevelopmental Genetics Unit, Center for Genomic Medicine, Massachusetts General Hospital, Boston, MA, United States; ^2^Department of Psychiatry, Massachusetts General Hospital/Harvard Medical School, Boston, MA, United States; ^3^Center on the Developing Child at Harvard University, Cambridge, MA, United States

**Keywords:** emotional neglect, social support, depression, childhood adversity, ALSPAC

## Abstract

**Introduction:** Childhood adversities have been shown to increase psychopathology risk, including depression. However, the specific impact of childhood emotional neglect on later depression has been understudied. Moreover, few studies have investigated relational protective factors that may offset the risk of depression for children who experienced emotional neglect. Analyzing data (*n* = 3,265) from the Avon Longitudinal Study of Parents and Children (ALSPAC) study, a longitudinal birth cohort of children born to pregnant women residing in Avon, UK from 1990 to 1992, we assessed the prospective relationship between childhood emotional neglect and depressive symptoms in late adolescence, and tested whether peer social support in mid-adolescence moderates this relationship.

**Methods:** Childhood emotional neglect, defined as the absence of parental attention and support, was measured across seven assessments from age 8 to 17.5. Peer social support was measured at age 15. Depressive symptoms were measured at age 18. We analyzed the associations between emotional neglect and depressive symptoms, and between peer support and depressive symptoms, and also tested interactive effects of peer support on the association between emotional neglect and depressive symptoms.

**Results:** Higher levels of emotional neglect were associated with increased depressive symptoms at 18. Conversely, strong peer social support was associated with reduced depressive symptoms, though no significant interaction with emotional neglect was detected.

**Conclusion:** Although childhood emotional neglect is a risk factor for later depression, our results suggest that strong peer social support at age 15 may generally reduce the risk of depressive symptoms by the time children reach late adolescence. Fostering strong peer support in youth may help offset depression risk for all youth, even among those who have experienced emotional neglect.

## Introduction

At least one in five youth in the United States, the United Kingdom, and other developed countries (1–3) experience childhood adversities such as exposure to abuse, neglect, or other major life stressors, which can considerably impact their socio-emotional, cognitive, and physical development, while simultaneously increasing the long-term risk of mental health difficulties (4, 5). In particular, exposure to childhood adversities can more than double the risk of depression in later childhood and adulthood and is also associated with increased suicide rates in adulthood (6–8).

One form of childhood adversity that has remained relatively understudied in its long-term mental health impact is emotional neglect (9). Emotional neglect can be defined as caregivers' persistent disregard of children's emotional needs, including failure to provide comfort when a child becomes scared or distressed (10). Importantly, exposure to childhood emotional neglect is associated with poor developmental outcomes such as increased maladaptive behaviors and lower self-esteem (5, 10), as well as psychopathology in adulthood (11). However, few studies have focused specifically on childhood emotional neglect with regards to its impact on depression risk in late adolescence, a vulnerable developmental stage when the risk of developing depression is particularly high (12) and when the prevalence of depression has increased substantially over the past decade, according to national estimates from the United States (13).

Although childhood adversities such as emotional neglect can exert lasting consequences on mental health outcomes during development, these deleterious effects may be buffered by positive experiences. Understanding potential protective factors, particularly those that could be intervened upon earlier in development, could foster strategies to support the long-term mental health of youth facing adversities such as emotional neglect. While the protective factors that may help mitigate the effects of childhood emotional neglect remain largely unknown, one particularly promising factor that may buffer the negative effects of childhood emotional neglect on depression risk is social support (14). Social support, defined as the extent to which individuals may receive emotional or instrumental help from others, is a noteworthy predictor of children's positive psychosocial development such as increased self-esteem and confidence (15, 16). Strong social support from close others can protect against negative mental health outcomes such as depression, both in general (17) and following childhood adversities such as emotional, physical, and sexual abuse in childhood and adulthood (14, 18). Importantly, considerable research on childhood adversities has mostly focused on the supportive role of family members (19, 20); however, children specifically experiencing emotional neglect at home may instead need to develop positive and strong relationships outside the home, with their peers (21).

Promising observational data suggest that strong peer social support is inversely associated with depressive symptoms among individuals with a history of childhood adversities (22) and that higher perceived peer social support may weaken the association between reported childhood adversities and depressive symptoms in adulthood (14). However, these studies have been conducted retrospectively in adults, who reported on their current peer support rather than support they may have received as children. By contrast, less is known about protective effects of peer social support during adolescence. Given that adolescence is a crucial time for peer bonding and relationship development (23), peer social support during this developmental period may be an important and intervenable protective factor that could promote long-term wellbeing and resilience to depression despite childhood adversities experienced at home. Importantly, the extent to which peer social support observed in mid-adolescence can prospectively influence depression risk and moderate the potentially deleterious effects of childhood emotional neglect remains unknown.

To address these knowledge gaps, we analyzed data from the Avon Longitudinal Study of Parents and Children (ALSPAC), a longitudinal birth cohort that followed mothers and their children residing in Avon, UK from before birth through the child's mid-twenties (24, 25). This longitudinal cohort provides the unique opportunity to characterize childhood emotional neglect across multiple points during childhood and prospectively capture peer social support during adolescence in relation to later depression risk. Our study was informed by theories on emotional and behavioral development in children (14, 26, 27), which suggest childhood adversities, including deprivation from normative caregiver inputs, may increase risk for poor self-esteem, altered reward processing, and depression across development. Our study aimed to: (1) assess the relationship between childhood emotional neglect and depressive symptoms in adolescence, (2) investigate the association between peer social support and adolescent depressive symptoms, as well as (3) test the combined and interactive effects of peer social support and childhood emotional neglect on adolescent depressive symptoms. Given previous research linking childhood adversities to negative mental health outcomes and the role of increased social support as a protective factor for poor health outcomes in adolescence, we hypothesized that childhood emotional neglect would be associated with increased depressive symptoms and that strong peer social support would be associated with lower depressive symptoms. Further, we hypothesized that peer support would moderate the relationship between childhood emotional neglect and depressive symptoms, such that the association between emotional neglect and later depressive symptoms would be attenuated in children who experienced higher peer support in adolescence.

## Materials and Methods

Women residing in Avon, UK, a city in the southwest of England, with expected delivery dates from April 1991 to December 1992 who were willing to participate in the study were recruited into ALSPAC (*n* = 14,541 pregnancies) (24, 25, 28). Of the women who gave birth, there were 14,062 live births; the children alive at 1 year of age (*n* = 13,988) were enrolled in the study. An additional 913 children were enrolled in the study after age 7, resulting in a total sample size of 14,901 children that were alive at 1 year of age. Compared to UK mothers and Avon mothers, ALSPAC mothers were more likely to be White, married, and own a house (24). Additional ALSPAC information can be found using a searchable data dictionary and variable search tool on the ALSPAC website: http://www.bristol.ac.uk/alspac/researchers/our-data/. Ethical approval for the study was obtained from the ALSPAC Ethics and Law Committee and the Local Research Ethics Committees. Informed consent for the use of data collected via questionnaires and clinics was obtained from participants following the recommendations of the ALSPAC Ethics and Law Committee at the time.

### Measures

#### Childhood Emotional Neglect by Parents

We used repeated measures of parental attention and monitoring to create our emotional neglect variable; previous studies have shown that repeated measures of child maltreatment can minimize recall biases (29). Our emotional neglect variable is consistent with definitions from the World Health Organization ACE International Questionnaire and previous published studies from the ALSPAC cohort that have used a similar approach to define emotional neglect based on low levels of parental attention and monitoring reported by children over time (10, 30). Specifically, children prospectively reported their experiences of parental attention and monitoring at eight separate assessments from 8 to 17.5 years of age (97, 116, 150, 162, 168, 186, 192, 210 months). Participants were asked to respond to statements such as “How often do your caretakers take the time to listen to you when you talk about what happened during your free time?” and “How often do your caretakers ask what happened at school, on a normal school day?” Response options were based on either a 4-point or 5-point Likert scale (more details for each response option can be found in Supplementary Table 1).

In line with previous approaches (10), each of these 23 items was then dichotomized with the most extreme response for each item coded as a 1 (exposed to emotional neglect), while all other responses were given a 0 (unexposed to emotional neglect). Conceptually, we determined that only the complete absence of monitoring and attention (e.g., response options indicated as “none”) should be taken to reflect a problematic situation of parental neglect, rather than this construct of neglect existing on a gradient opposite to parental monitoring. Therefore, and similar to studies elsewhere, we only took the most extreme response for each item as contributing to a total score reflecting emotional neglect. Previous research in the ALSPAC cohort has operationalized emotional neglect in a similar binary fashion based on the reported absence of parental attention and monitoring (10). See Supplementary Table 1 for the variables used and criteria. The 23 items that resulted from this binary classification strategy were summed to create one total quantitative score for emotional neglect, with values ranging from 0 to 14 in our study (maximum possible score of 23). There was moderately-strong internal consistency reliability for the 23 parental monitoring items used to construct the emotional neglect variable in our sample, a = 0.79. Based on a histogram of this total quantitative score, participants were then categorized into one of four levels of exposure to emotional neglect: none (total score = 0); mild (total score = 1); moderate (total score = 2); high (total score = 3 or more).

#### Perceived Peer Social Support

Perceived peer social support was reported by children at age 15 with the five-item shortened version of the Cambridge Hormones and Moods Project Friendship questionnaire (31). We chose to focus on children's perceived social support at age 15 because acceptance and support by peers at this age is a strong predictor of self-worth and self-esteem (32). This five-item friendship questionnaire asks youth respondents to report on their perceived peer social support using a 4-point Likert scale, with each item ranging from 0 to 3. Items included: “Overall how happy are you with your friends (3 = most of the time, 2 = sometimes, 1 = not often, 0 = not at all),” “Do your friends understand you (3 = most of the time, 2 = sometimes, 1 = not often, 0 = not at all),” “Do you talk to your friends about problems (3 = most of the time, 2 = sometimes, 1 = not often, 0 = not at all),” “Are you happy with the number of friends you've got (3 = very happy, 2 = quite happy, 1 = quite unhappy, 0 = unhappy),” and “Do you see your friends outside of school (3 = almost every day, 2 = at least once per week, 1 = less than once per week, 0 = hardly ever).” The five items were summed to create one total score ranging from 0 to 15, with greater scores indicating greater peer social support, as scored in previous research (33). The mean inter-item correlation for the five-item perceived social support questionnaire was modest (*r* = 0.22), which is similar to previous research findings on this measure (34).

#### Depressive Symptoms

Depressive symptoms were reported by adolescents at age 18 using the Short Mood and Feelings Questionnaire (SMFQ); (35, 36). The SMFQ includes 13 questions asking respondents to report on depressive symptoms in the past 2 weeks using a 3-point Likert scale, ranging from 0 to 2 (0 = not true, 1 = sometimes true, 2 = true). All items were summed to create one total score, ranging from 0 to 26 with higher scores indicating increased symptoms of depression. Some of the items are related to emotions, such as “I felt miserable or unhappy” or “I felt I was not good anymore,” whereas others were related to cognition and thoughts, such as “I found it hard to think properly and concentrate” or “I thought nobody really loved me.” We found strong internal consistency for SMFQ in our sample, α = 0.91. The SMFQ has previously shown strong validity and has been used in prior research with ALSPAC data as well as many other studies (37).

#### Covariates

All models included the following covariates: the child's sex, race, and mother's age at child's birth, number of previous pregnancies, marital status, home ownership status, and education attainment at the time she gave birth. These covariates were selected as key sociodemographic characteristics from our work and prior literature that could potentially influence the relationship between exposure to emotional neglect and later depressive symptoms (38, 39). We also included two potential childhood adversities—mother's psychopathology when the child was 8 months old, and being born into a one-parent household—as additional covariates in a sensitivity analysis.

### Statistical Analyses

#### Imputation

To prevent the loss of information, improve the statistical power to detect associations in the data, and reduce bias in our effect estimates, we performed multiple imputation using chained questions (MICE package in R, version 3.13.0) for missing data (40). Multiple imputation using chained questions is an iterative procedure for computing missing data. It operates by systematically calculating the missing values for each variable using a regression model containing the non-missing data from other specified variables available in the dataset. For our MICE procedure, we implemented this iterative process 25 times for each of the 20 imputed datasets we created. Each of the 20 datasets contained all ALSPAC participants with SMFQ scores available at age 18 (*n* = 3,263). We then performed our inference using the *pooled* estimates from those 20 imputed datasets (41).

All confounders and exposures were imputed for in the imputation model. Data on the child's depressive symptoms at age 18 as well as variables pertaining to the child's friendships throughout adolescence, a strong predictor of social support levels (42), were included in the imputation procedure to provide additional information in an effort to yield more accurate estimates for missing covariate and exposure data.

#### Regression Models

We ran three linear regression models. Model 1 tested the association between levels of childhood emotional neglect and depressive symptom scores at 18. Model 2 added to Model 1 by including peer social support, allowing us to assess the effect of both emotional neglect and levels of peer social support on depressive symptoms at age 18. Using an interaction term between childhood neglect and the levels of social support, Model 3 added to Model 2 by assessing whether social support was an effect modifier of the relationship between childhood emotional neglect and depressive symptoms. To increase interpretability of the results, we standardized the SMFQ depression outcome variable with a mean of 0 and *SD* of 1, such that beta values for each of the independent variables in the model reflected standard deviation changes in the depressive symptoms. All models were adjusted for the covariates as described in the Measures (e.g., home ownership status, child sex, race).

## Results

### Sample Description

Our analytic sample was composed of 3,263 singleton children with complete outcome data on depressive symptoms at age 18 (Table 1). Demographics of the complete case sample prior to imputation are shown in Table 1. Of the 3,263 participants, 64.4% identified as female, 96.1% identified as white, and most were first- or second-born children (48.9%, 34.8%, respectively). Eighty-nine percent of the mothers were 20–35 years old at the child's birth, 22.5% had a University degree or above, 83.9% were married, and 85.1% had a mortgage or owned a home.

**Table 1:** Baseline characteristics of participants in the analytic sample (*n* = 3,263).

	**Analytic sample^a^ = 3,263**	
**Variable**	***N* (%)**	**Missing (%)**
Child's sex (female)	2,100 (64.4)	0.0%
Child's race (White)	2,905 (96.1)	7.4%
Number of previous pregnancies		
0	1,494 (48.9)	6.4%
1	1,064 (34.8)	
2	375 (12.3)	
3+	121 (4.0)	
Mother's age at child's birth		
Ages 15–19	45 (1.4)	4.3%
Ages 20–35	2,763 (88.5)	
Ages 35+	315 (10.1)	
Mother's marital status		
Never married	362 (11.8)	5.6%
Widowed/divorced/separated	133 (4.3)	
Married	2,585 (83.9)	
Home ownership		
Mortgage/own home	2,590 (85.1)	6.8%
Rent home	370 (12.2)	
Other	82 (2.7)	
Maternal education^b^		
Less than O-level	529 (17.3)	6.5%
O-level	981 (32.2)	
A-level	856 (28.1)	
Degree or above	685 (22.5)	

a *Analytic sample pre-imputation, includes some missing data for demographic variables*.

b *Highest level of education of mother at 32-weeks gestation: O-level (“ordinary-level” exams obtained by students at age 16 in the UK), A-Level (“advanced-level” exams obtained by students at age 18 in the UK), Degree or above (obtained University degree or higher)*.

Total depressive symptom scores, measured using the SMFQ, ranged from 0 to 26 (*M* = 6.80, *SD* = 5.89) (Table 2). Nearly 9% of children in our analytic sample could be classified as experiencing high exposure to emotional neglect, 10% with moderate exposure, 18% with low exposure, and 63% with no exposure. The distribution of the peer social support measure was left-skewed toward higher scores (*M* = 12.94, *SD* = 1.69, ranged from 0 to 15), with individuals tending to endorse relatively high levels of peer social support.

**Table 2:** Descriptive statistics for depressive symptoms, social support, and childhood emotional neglect variables in the analytic sample (*n* = 3,263).

**Variable**	**Analytic sample^a^** **=** **3,263**			
	***N* (%)**	***Mean* (*SD*)**	***Min***	***Max***
Depressive symptoms	–	6.80 (5.89)	0	26
Social support	–	12.94 (1.69)	3	15
Severity of emotional neglect				
None	2056 (63)			
Mild	587 (18)			
Moderate	326 (10)			
High	294 (9)			

a *Analytic sample pre-imputation, includes some missing data for demographic variables*.

### Association Between Childhood Emotional Neglect and Depressive Symptoms at 18

To determine the influence of emotional neglect on depression risk, we next examined the relationship between childhood emotional neglect and depressive symptoms at age 18. Mild, moderate, and high levels of exposure to childhood emotional neglect, compared to those with no exposure, were significantly associated with increased depressive symptoms at age 18 (β = 0.15, *p* < 0.01; β = 0.31, *p* < 0.01; β = 0.38, *p* < 0.01) (Table 3, Model 1), with a Model 1 *R*^2^ of 0.06.

**Table 3:** Results of linear regression analysis assessing the association between degree of severity of childhood emotional neglect on depressive symptoms at age 18, adjusting for covariates.

	**Model 1*^a^***				**Model 2*^a^***				**Model 3*^a^***			
	**β**	***SE***	***P*-value**	**95% CI**	**β**	***SE***	***P*-value**	**95% CI**	**β**	***SE***	***P*-value**	**95% CI**
**Severity of emotional neglect**												
None	Ref.				Ref.				Ref.			
Mild	0.15	0.06	<0.01^*^	(0.04, 0.27)	0.14	0.06	<0.01^*^	(0.03, 0.25)	−0.27	0.46	0.55	(−0.35, 0.88)
Moderate	0.31	0.07	<0.01^*^	(0.16, 0.46)	0.29	0.07	<0.01^*^	(0.15, 0.44)	−0.08	0.59	0.89	(−1.08, 1.24)
High	0.38	0.08	<0.01^*^	(0.22, 0.53)	0.36	0.08	<0.01^*^	(0.20, 0.51)	−0.78	0.60	0.19	(−1.96, 0.40)
*Social support*					−0.05	0.01	<0.01^*^	(−0.08, −0.03)	−0.07	0.02	<0.01^*^	(−0.10, −0.04)
**Interaction term**												
None × social support									Ref.			
Mild × social support									0.03	0.04	0.37	(−0.04, 0.10)
Moderate × social support									0.02	0.05	0.72	(−0.07, 0.10)
High × social support									0.09	0.05	0.06	(−0.01, 0.18)

a *Covariates included child's sex, child's race, mother's number of previous pregnancies, mother's age at child's birth, mother's marital status, maternal homeownership, maternal education, maternal psychopathology, and one-parent household*.

* *α < 0.001*.

### Association Between Emotional Neglect, Peer Support, and Depressive Symptoms at 18

We next examined the role of peer social support and emotional neglect on depressive symptoms by including both peer social support and emotional neglect as independent main effects in the same model. The overall Model 2 had a *R*^2^ of 0.07. Here, we found that mild, moderate, and high levels of exposure to emotional neglect were still associated with significantly higher depressive symptoms at age 18 compared to no exposure (β = 0.14, *p* = 0.01; β = 0.29, *p* < 0.01; β = 0.36, *p* < 0.01, respectively) (Table 3, Model 2). We also found a significant negative association between perceived peer social support and depressive symptoms (β = −0.05, *p* < 0.01) indicating that as perceived peer social support increases, depressive symptoms decrease, even after adjusting for childhood emotional neglect (Table 3, Model 2). This adjusted association of peer social support is notable because preliminary analyses indicated that continuous social support and emotional neglect scores were negatively correlated to a statistically significant but modest extent (*r*_*s*_ = −0.09, *p* = 0.01 in non-imputed sample).

### Interaction Between Peer Support and Emotional Neglect on Depressive Symptoms at 18

Finally, we examined the statistical interaction between social support and emotional neglect to determine whether peer social support moderated the relationship between emotional neglect and depressive symptoms. The overall Model 3 had a *R*^2^ of 0.07. We did not detect any significant statistical interaction between levels of emotional neglect and social support on depressive symptoms (mild, *p* = 0.55, moderate, *p* = 0.89, high *p* = 0.19 at the α = 0.05 level) (Table 3, Model 3). In this model, social support maintained a significant independent main effect with fewer depressive symptoms (β = −0.07, *p* < 0.01), while emotional neglect no longer showed a significant main effect (Table 3, Model 3). Notably, the direction of effect and pattern of results in all three models remained consistent even in sensitivity analyses adjusting for other potential early adversities (i.e., maternal psychopathology, one-parent household).

## Discussion

In this prospective study, we found that childhood emotional neglect was significantly associated with depressive symptoms in late adolescence, whereas stronger peer social support in mid-adolescence was associated with reduced depressive symptoms, even after accounting for emotional neglect. Peer support did not show a statistical interaction with emotional neglect, suggesting that the protective effects of peer social support on depression held both for children who experienced emotional neglect as well as those who did not.

To date, limited research has focused specifically on the influences of childhood emotional neglect on mental health outcomes. Emotional neglect is less overt than other forms of maltreatment, such as physical abuse, making it a relatively challenging construct to assess (5, 9, 43). Informed by similar approaches in prior literature (8), we established a working empirical definition in which the presence of low levels of parental attention, support, and monitoring reported by children over time was aggregated into a quantitative index of emotional neglect exposure, and then categorized to reflect severity of this exposure. Per this definition, 19% of our study sample had experienced at least moderate to high levels of emotional neglect. This highlights the prevalence of emotional neglect experiences during childhood in a population-based birth cohort and the need to understand its long-term mental health consequences. Prior studies focusing on a broader range of childhood adversities have suggested that emotional forms of abuse and neglect have particularly strong associations with depression relative to other forms (e.g., physical abuse, sexual abuse, and physical neglect) (5). Our study focused specifically on emotional neglect provides further prospective support for its role as a risk factor for later depression. Further understanding about the mechanisms of this association—that is, how emotional neglect translates into depression risk—is needed. Some research has shown that emotional neglect often co-occurs with other forms of childhood maltreatment (9), and so it may be a marker of other adverse experiences.

In our study, we found that peer social support in mid-adolescence was significantly associated with reduced depressive symptoms in late adolescence. Our findings are similar to previous research in adult samples reporting that childhood adversity (including emotional neglect) was associated with later depression and that social support was linked to a decrease in adulthood depressive symptoms (14). While this result aligns with a robust and growing evidence base linking peer social support and depressive outcomes across the life course (44), our study contributes further evidence that peer social support is protectively associated with depressive symptoms in adolescence, a key time period for the emergence of depression, even after adjusting for exposure to childhood emotional neglect. Strong peer social support has been linked to academic achievement, positive self-worth, and quality of life across development (20, 45, 46). Importantly, our study suggests that strong perceived support from peers during mid-adolescence, a time when youth increasingly rely on relationships outside the home (47), is generally beneficial and could even offset the negative mental health impacts of emotional neglect from caregivers. Children with stronger peer support may receive the benefits of sharing similar experiences, distraction, and positive interactions that in turn, lead to lower likelihood of depression. Future studies should examine additional protective factors related to childhood adversity, such as participating in extracurricular activities and satisfaction with school, that could decrease depressive symptoms even in the context of emotional neglect (48).

Prior research has also suggested there may be specific developmental stages or sensitive periods when children are not just more vulnerable to developing psychopathology, but may also be more responsive to interventions (49). While we selected mid-adolescence based on developmental theory and available data, future research should aim to empirically determine whether there are specific sensitive periods in development where the strengthening of peer social support may be particularly helpful in offsetting risk for depression. Moreover, whether other forms of social support that may be available to youth, such as teacher and broader community support, can also mitigate depression risk in children exposed to emotional neglect is also a topic requiring further investigation.

In this study, we did not observe a statistical interaction between peer social support in mid-adolescence and emotional neglect, and so cannot conclude that peer support specifically buffers the effect of emotional neglect. This finding is somewhat contrary to retrospective studies in adult cohorts, which have found that peer social support, at least in adulthood, may buffer the effects of childhood adversities (e.g., emotional neglect, physical abuse, household substance abuse) on depressive symptoms (14). It is possible that peer support, or other forms of social support, only show an interactive buffering effect with other forms of childhood adversities and not emotional neglect. The absence of a significant statistical interaction may also be due to a modest sample size for testing statistical interactions, although this suggests that even if an interaction truly does exist, its magnitude is likely to be small in nature. However, we found that the effects of peer social support persisted even after adjusting for emotional neglect and other potential confounders, indicating that the protective influences of peer social support apply even in the context of emotional neglect exposure. As such, all children, even those who have experienced significant neglect from their caregivers, may experience a reduction in depression risk if they have strong relationships with their peers.

## Limitations

One potential limitation of this study is our empirical definition of emotional neglect. Although we relied on existing approaches in defining emotional neglect on the basis of extreme low responses to measures that assess parental monitoring, support, and attention (10), future work may benefit from refining assessments for emotional neglect that specifically measure the extent of caregiver emotional responsiveness vs. unavailability to the child's emotional and psychological needs. Including more direct and comprehensive questions would likely result in a more accurate measurement of this important construct (10). Future studies should address cultural differences when designing a measure for emotional neglect, which may influence self-reporting and perceptions of maltreatment (9). While similar overall, there are some sociodemographic differences between mothers and children from the ALSPAC cohort in comparison to the UK population (e.g., race distribution, socioeconomic factors) that may limit the generalizability of our findings. The moderate sample size and the homogenous population were other limitations of this study, which may have limited our ability to detect significant interactive associations and extend interpretation of our findings to broader contexts, respectively. Further research might also assess the influence of peer social support on depression risk for children exposed to emotional neglect and other forms of maltreatment in more racially and ethnically diverse populations. Although our study focused primarily on emotional neglect independent from other childhood adversities and also accounted for two other childhood adversity exposures (i.e., maternal psychopathology, one-parent household), future studies may incorporate additional ACEs and contextual exposures as potential covariates in the model, because particularly for some groups childhood adversities often co-occur. Finally, our study focused on observational associations. Although our prospective design strengthens the inference that peer social support may indeed affect future risk of depression, rather than the other way around, adolescents who are vulnerable to depression may also have difficulties with building strong peer relationships for example, due to traits such as interpersonal sensitivity (50). Depression could also lead to decreases in peer support (51). More longitudinal studies with prospective measures of both depression and peer support across time may allow us to better disentangle these temporal relationships. Moreover, potential recall bias may have influenced participant's accurate self-reporting of childhood emotional neglect, whether through underreporting or overreporting (29).

## Conclusion

Our findings suggest that strong social support at age 15 may be a protective factor linked to reduced depressive symptoms in youth, even among those who have been neglected emotionally. Schools, caretakers, and health professionals should be alert to the identification of emotional neglect because children who are affected are likely to be at higher risk for depressive symptoms later in life. These findings may well lead to clinicians and schools enhancing opportunities for socially supportive peer interventions that optimize mental health outcomes for vulnerable children.

## Data Availability Statement

Publicly available datasets were analyzed in this study. This data can be found here: http://www.bristol.ac.uk/alspac/researchers/our-data/.

## Ethics Statement

The studies involving human participants were reviewed and approved by ALSPAC Ethics and Law Committee and the Local Research Ethics Committees. Written informed consent to participate in this study was provided by the participants' legal guardian/next of kin.

## Author Contributions

EG, KC, AL, BS, and ED contributed to conception and design of the study. All authors contributed to the article and approved the submitted version.

## Author Disclaimer

The content is solely the responsibility of the authors and does not necessarily represent the official views of the National Institutes of Health.

## Conflict of Interest

The authors declare that the research was conducted in the absence of any commercial or financial relationships that could be construed as a potential conflict of interest.

## Publisher's Note

All claims expressed in this article are solely those of the authors and do not necessarily represent those of their affiliated organizations, or those of the publisher, the editors and the reviewers. Any product that may be evaluated in this article, or claim that may be made by its manufacturer, is not guaranteed or endorsed by the publisher.
