# Supplementary material for: Childhood Emotional Neglect and Adolescent Depression: Assessing the Protective Role of Peer Social Support in a Longitudinal Birth Cohort
Source: Front Psychiatry. 2021 Aug 9;12:681176. doi: 10.3389/fpsyt.2021.681176 (PMC8381469; doi:10.3389/fpsyt.2021.681176)
Supplement: Supplementary file 1 [file Data_Sheet_1.PDF]

Supplementary Material

Supplementary Table 1.

ALSPAC variables used from adverse childhood experiences (ACEs) for defining emotional neglect and recoding of emotional neglect variables.

Description	Recoding	Dichotomizing strategy	Age reported
Frequency child feels left out of things	1=always, 2=sometimes, 3=often, 4=never	1=always	97m
Study child is understood by parent(s)	1=not true, 2=mostly untrue, 3=partly true, 4=mostly true, 5=true	1=not true	116m
Frequency carers ask teenager what happened in their free time	1=never, 2=hardly ever, 3=sometimes, 4=most of time, 5=always	1=never	12.5yrs
Frequency past month carers started conversation about teenager's spare time	1=never, 2=hardly ever, 3=sometimes, 4=most of time, 5=always	1=never	12.5yrs
Frequency carers take time to listen, when teenager talks about what happened in their free time	1=never, 2=hardly ever, 3=sometimes, 4=most of time, 5=always	1=never	12.5yrs
Frequency carers know who teenager's friends are, outside of school	1=never, 2=hardly ever, 3=sometimes, 4=most of time, 5=always	1=never	12.5yrs
Frequency carers ask teenager what happened at school, on a normal day school day	1=never, 2=hardly ever, 3=sometimes, 4=most of time, 5=always	1=never	12.5yrs
Frequency carers ask teenager what happened in free time	1=never, 2=hardly ever, 3=sometimes, 4=most of time, 5=always	1=never	13.5yrs
Frequency past month carers started conversation about teenager's spare time	1=never, 2=hardly ever, 3=sometimes, 4=most of time, 5=always	1=never	13.5yrs
Frequency carers take time to listen, when teenager talks about what happened in free time	1=never, 2=hardly ever, 3=sometimes, 4=most of time, 5=always	1=never	13.5yrs
Frequency carers know who teenagers friends are, outside of school	1=never, 2=hardly ever, 3=sometimes, 4=most of time, 5=always	1=never	13.5yrs
Frequency carers ask teenager what has happened at school, on normal school day	1=never, 2=hardly ever, 3=sometimes, 4=most of time, 5=always	1=never	13.5yrs
YP spoke to mother or female adult about how things are going with friends in the last month	4=most days, 3=sometimes, 2=rarely, 1=never	1=never	14yrs

YP spoke to father or male adult about how things are going with friends in the last month	4=most days, 3=sometimes, 2=rarely, 1=never	1=never	14yrs
YP spoke to mother or female adult about how things are going at school in the last month	4=most days, 3=sometimes, 2=rarely, 1=never	1=never	14yrs
YP spoke to father or male adult about how things are going at school in the last month	4=most days, 3=sometimes, 2=rarely, 1=never	1=never	14yrs
Frequency carer asks YP what they did in their free time	1=never, 2=hardly ever, 3=sometimes, 4=most of time, 5=always	1=never	15.5yrs
Frequency carer started conversation about YPs free time, in last month	1=never, 2=hardly ever, 3=sometimes, 4=most of time, 5=always	1=never	15.5yrs
Frequency carer listens to YP, when they talk about what they they did in their free time	1=never, 2=hardly ever, 3=sometimes, 4=most of time, 5=always	1=never	15.5yrs
Frequency carer knows the friends that YP has outside school	1=hardly ever, 2=sometimes, 3=most of time, 4=always	1=hardly ever	15.5yrs
Frequency carer asks YP about what happened at school on normal school day	1=never, 2=hardly ever, 3=sometimes, 4=most of time, 5=always	1=never	15.5yrs
Frequency over last term parents have asked YP how they are getting on with different subjects at school	4=most days, 3=sometimes, 2=rarely, 1=never	1=never	16 yrs
How easy YP finds it to discuss their problems with anyone in their family	5=very easy, 4=quite difficult, 3=neutral, 2=quite difficult, 1=very difficult	1=very difficult	17.5yrs

Supplementary Table 2.

Perceived peer social support variables were measured by the 5-item shortened version of the Cambridge Hormones and Moods Project Friendship questionnaire in ALSPAC. These five variables were summed to create one total score for social support at age 15 and were used in imputation and statistical analyses.

Description	Recoding	Age reported
Teenager is happy with number of friends	3=Very happy, 2=Quite happy, 1=Quite unhappy, 0=Unhappy	15yrs
Frequency teenager sees friends out of school	3=almost every day, 2= at least once per week 1=less	15yrs

	than once per week, 0=hardly ever or never	
Teenager believes friends understand them	3=most of the time, 2=sometimes, 1=not often, 0=not at all	15yrs
Teenager talks about problems with friends	3=most of the time, 2=sometimes, 1=not often, 0=not at all	15yrs
Degree to which teenager is happy with his/her friends overall	3=most of the time, 2=sometimes, 1=not often, 0=not at all	15yrs
